# Treatment of Symptomatic Male Hypogonadism with New Oral Testosterone Therapies: A Comparative Review of Jatenzo, Tlando, and Kyzatrex

**DOI:** 10.3390/medicines13010001

**Published:** 2025-12-22

**Authors:** Samantha H. Rosen, Kian Asanad

**Affiliations:** Keck School of Medicine of USC, University of Southern California Institute of Urology, Los Angeles, CA 90089, USA; shrosen@usc.edu

**Keywords:** testosterone replacement therapy, male hypogonadism, oral androgen therapy, Jatenzo, Tlando, Kyzatrex

## Abstract

Symptomatic male hypogonadism, defined by low serum testosterone with associated clinical symptoms, is increasingly treated with testosterone replacement therapy. Traditional oral formulations were limited by hepatotoxicity and poor bioavailability, leading to reliance on injectable and transdermal routes. Recent advances in oral testosterone undecanoate formulations have introduced safer and more effective options. This review compares Jatenzo, Tlando, and Kyzatrex, highlighting their pharmacology, efficacy, safety, and clinical utility. Clinical trial data demonstrate restoration of eugonadal testosterone levels in most patients (80–88%), with shared risks including hypertension, polycythemia, and lipid changes. Differences in dosing regimens, titration requirements, and insurance coverage influence choice of therapy and patient adherence. Kyzatrex offers flexible titration and self-pay access, Tlando provides a fixed-dose regimen, and Jatenzo combines titratability with established clinical data. Collectively, these agents expand the therapeutic landscape of hypogonadism, offering effective, non-invasive alternatives that support individualized treatment strategies.

## 1. Introduction

Symptomatic male hypogonadism is a clinical condition defined by the combination of symptoms consistent with androgen deficiency and two separate morning low serum total testosterone concentration measurements less than 300 ng/dL. These symptoms often include reduced libido, erectile dysfunction, fatigue, loss of secondary sex characteristics, decreased muscle mass, anemia, and impaired physical performance. Notably, the Endocrine Society highlights that sexual symptoms, such as poor morning erections, decreased libido, and erectile dysfunction, are the most strongly associated with low testosterone levels and are the most reliable indicators of clinically significant hypogonadism in adult men [1].

Testosterone therapy (TTh) is indicated for men diagnosed with symptomatic hypogonadism, where both clinical symptoms and low testosterone levels are evident. However, testosterone therapy is not recommended for men with low testosterone levels in the absence of signs/symptoms, nor for those with reversible or functional causes of hypogonadism unless symptoms persist after addressing underlying factors [1,2]. For those who qualify, TTh aims to induce and maintain secondary sex characteristics, correct symptoms of androgen deficiency, and ultimately improve patients’ quality of life [1,3].

Over the years, testosterone therapies have evolved significantly. While transdermal, injectable, and implanted pellet formulations are readily available, an oral option for men has been more challenged. Traditional oral testosterone therapy faced significant challenges related to hepatotoxicity and poor bioavailability [4]. This is due to the fact that unmodified oral testosterone undergoes extensive first-pass hepatic metabolism, resulting in rapid inactivation and very low systemic absorption [5,6]. This necessitated the development of oral testosterone undecanoate, absorbed via the lymphatic system, a novel pathway which allows for effective oral delivery while minimizing hepatic risk [7]. This evolution in testosterone replacement therapies culminated in the approval of three recently developed oral testosterone agents, including Jatenzo, Tlando, and Kyzatrex which have been shown to restore serum testosterone to the eugonadal range in symptomatic hypogonadal men [8,9,10]. These oral agents come with varying dosing regiments: Kyzatrex and Jatenzo require dose titration, while Tlando offers a fixed-dose regimen [8,10,11]. All three therapies have demonstrated efficacy in achieving target testosterone levels, with safety monitoring focused on cardiovascular, hematologic, and prostate-related risks [12].

This review aims to provide a comprehensive comparison of the newly developed oral testosterone therapies, Jatenzo, Tlando, and Kyzatrex, focusing on their development, effectiveness, and clinical outcomes. By evaluating these therapies, we aim to clarify their respective roles in the management of symptomatic male hypogonadism, highlighting the benefits and challenges associated with each, and informing clinical decision making regarding their use in treatment plans.

## 2. Development of Oral Testosterone Therapies

TTh has undergone significant advancements over the years. Early oral formulations like methyltestosterone and fluoxymesterone faced major drawbacks, including poor bioavailability and hepatotoxicity caused by extensive first-pass metabolism in the liver. These 17 α-alkylated derivates, developed to bypass hepatic metabolism, were linked to significant liver toxicity, ranging from cholestatic jaundice and peliosis hepatitis to benign adenomas and hepatocellular carcinoma [10,13].

The risk was strongly dose- and duration-dependent. In one cohort of 60 patients receiving methyltestosterone (50 mg three times daily), 32% developed abnormal liver function tests and 63% had abnormal liver scans, particularly in those treated for more than one year [14]. Histopathology revealed early peliosis hepatitis, and in one case, hepatic adenoma. Case reports have described hepatic adenomas and hepatocellular carcinoma after 5-11 years of methyltestosterone therapy [15,16,17]. The FDA label for methyltestosterone explicitly warns of rare but serious adverse hepatic reactions, recommending close liver function monitoring [18]. Fluoxymesterone, another α-alkylated androgen, carries a similar risk profile, although less extensively studied. Its use has been discouraged due to reports of hepatic neoplasia and bile acid dysregulation [16,19,20]. However, mechanistically both agents promote oxidative stress and hepatocyte hyperplasia which explains their hepatotoxic profile.

Because of these limitations, the use of methyltestosterone and fluoxymesterone is now infrequent and generally limited to circumstances where the potential benefits outweigh the risks, while always carefully monitoring hepatic function. Their toxicity drove the development of alternative delivery systems, such as injectable esters and transdermal preparations. However, these methods came with their own set of challenges, including fluctuating testosterone levels and difficulties with patient adherence [5,21].

The development of oral testosterone undecanoate marked a significant improvement in TTh and occupies a unique position in the evolutionary timeline of testosterone therapies. Oral TU is the first orally bioavailable formulation with a safety profile distinct from older oral androgens discussed above. Introduced in the 1970s, oral TU leveraged lymphatic absorption, bypassing the portal circulation and reducing first-pass hepatic metabolism and associated hepatotoxicity [5,10].

**Figure 1:**
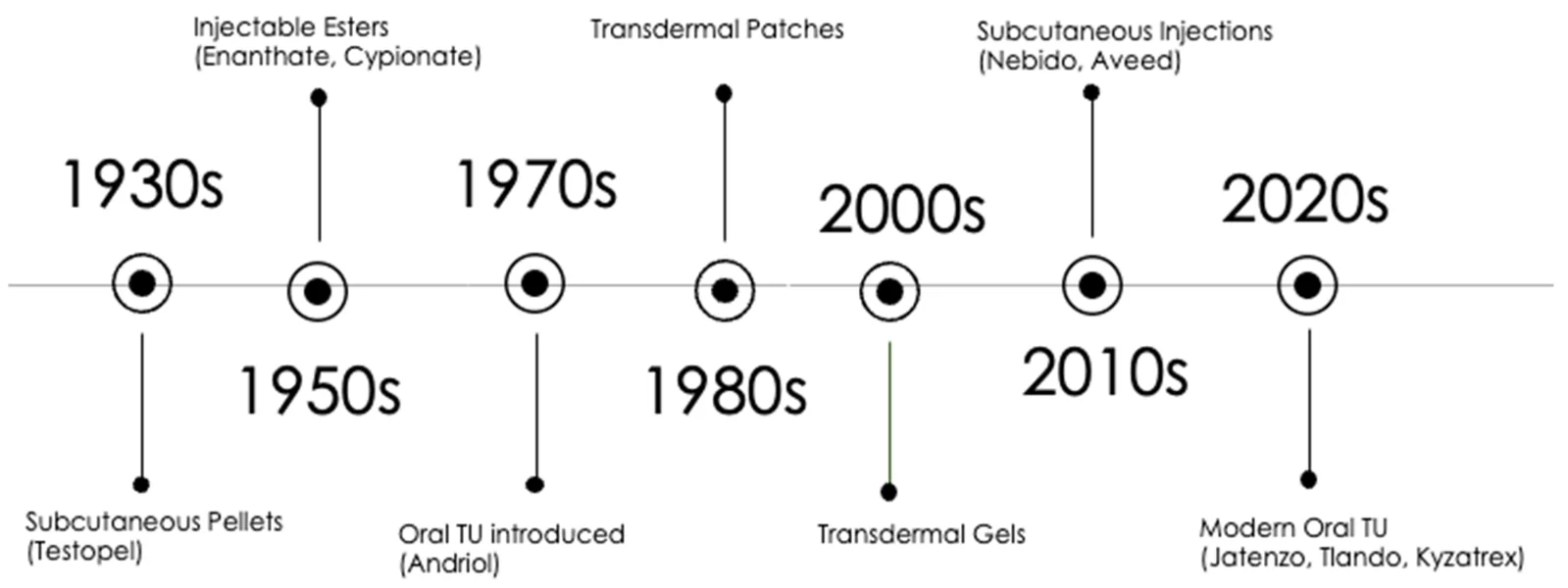
Timeline of testosterone therapy development.

Prior to this innovation, testosterone therapy had evolved through several delivery routes (Figure 1). Subcutaneous pellets (e.g., Testopel), developed in the 1930s, provided sustained release over 3–6 months but required minor surgical implantation [22]. Injectable testosterone esters (enanthate, cypionate), introduced in the 1950s, became widely adopted, though their pharmacokinetics produced supraphysiologic peaks and subtherapeutic troughs, leading to symptomatic fluctuations [5,6,19]. Transdermal preparations, including patches (1980s) and gels (2000s), provided more physiologic testosterone profiles and greater convenience, though with risks of skin irritation and inadvertent transfer [23,24]. More recently, long-acting injectable TU (e.g., Nebido, Aveed) has been developed, offering more stable serum levels with quarterly dosing and improved adherence [5,6,25]. In parallel, subcutaneous injections gained traction due to ease of administration and comparable pharmacokinetics compared to intramuscular injections [26].

Modern oral TU formulations represent a culmination of these developments. A key breakthrough came with the self-emulsifying drug delivery system (SEDDS), which combines hydrophilic and lipophilic components to solubilize testosterone undecanoate in the gut and promote intestinal lymphatic absorption independent of very high dietary fat intake [27,28]. Clinical trials have shown that these newer oral formulations effectively restore testosterone to eugonadal levels in men with symptomatic hypogonadism, with efficacy and safety comparable to non-oral routes [11].

When administered with dietary fat, oral testosterone undecanoate is absorbed through the intestinal lymphatics via incorporation into chylomicrons, resulting in more stable serum testosterone concentrations and a safety profile free from hepatic complications [10,29]. However, early testosterone undecanoate products such as Andriol (approved in many countries but not in the United States) relied heavily on high-fat meals for absorption, resulting in considerable intra- and inter-patient variability, frequent dosing requirements, and occasional gastrointestinal or hepatic adverse effects [6,10,30].

These advances have bridged the gap between the convenience of oral administration and safety and efficacy of parenteral and transdermal therapies. These new testosterone undecanoate options join the modern therapeutic landscape for symptomatic hypogonadism as a convenient, effective, and safe alternative that improves patient choice and adherence.

### 2.1. Jatenzo

Jatenzo is the first FDA approved oral testosterone undecanoate in SEDDS form indicated for TTh in adult males with confirmed primary or hypogonadotropic hypogonadism. The recommended starting dose is 237 mg orally twice daily with food, titrated between 158 mg and 396 mg twice daily based on serum testosterone levels measured 6 h after the morning dose.

The pivotal inTUne open-label trial (CLAR-15012) enrolled 166 hypogonadal men aged 18–75 years (mean age 56.2, 87% White) for an initial 4-month study period, with titration to maintain eugonadal serum testosterone concentrations. All patients were started on 237 mg twice daily, then titrated to 158 mg, 198 mg, 316 mg, or 396 mg twice daily [31,32]. At the 4-month primary endpoint of this trial, 87.3% of men in the oral testosterone undecanoate arm achieved serum testosterone levels within the eugonadal range [11]. Separately, in the CLAR-09007 program, a 12-month extension study was available to 129 eligible participants; 86 enrolled and 69 completed 24 months of uninterrupted therapy, with efficacy sustained over this 2-year period [33]. Beyond biochemical efficacy, Jatenzo demonstrated improvements in psychosexual function, measured by the Psychosexual Daily Questionnaire, a prespecified secondary endpoint, with gains in sexual activity, desire, and function sustained through 24 months in the extension program [33].

The safety profile includes secondary polycythemia, peripheral edema, and dyslipidemia [31]. A distinctive concern is blood pressure elevation, causing mean systolic BP increase by 3–6 mm Hg, with greater rises in men with pre-existing hypertension, and 7.2% of participants requiring initiation or escalation of antihypertensive therapy during the study [33]. Under current labeling, blood pressure elevation is a Warning and Precaution rather than a boxed warning, Jatenzo remains not recommended for men with uncontrolled hypertension, and regular monitoring of hematocrit, blood pressure, PSA, and lipids is required [31].

Important limitations of the available data include the open-label design and absence of placebo or comparator arms, which reduce internal validity and preclude precise attribution of efficacy or safety signals to Jatenzo alone. No large randomized, placebo-controlled trials directly compare Jatenzo to injectable or transdermal formulations, leaving its relative efficacy uncertain.

The pivotal trial (CLAR-15012; NCT02722278) was a randomized, active-controlled, open-label study in which patients were assigned in a 3:1 ratio to oral testosterone undecanoate (*n* = 166) or a 2% topical testosterone solution (*n* = 55) as active comparator [34]. Important limitations of the available data include the open-label design, the absence of a placebo arm, and the fact that efficacy was summarized separately by treatment group without formal statistical comparison between arms, which reduce internal validity and preclude precise attribution of efficacy or safety signals to Jatenzo alone. No large randomized, placebo-controlled trials directly compare Jatenzo to injectable or transdermal formulations, leaving its relative efficacy uncertain.

### 2.2. Tlando

Tlando is a once-daily oral testosterone undecanoate option for testosterone replacement in adult men with confirmed primary or hypogonadotropic hypogonadism. Unlike Jatenzo, which requires titration, Tlando is administered as a fixed dose of 225 mg twice daily with food, eliminating the need for individualized dose adjustments [9].

The efficacy and safety of Tlando was evaluated in a multicenter, open-label, single-arm study (Study 16-002, NCT03242590), that enrolled 95 hypogonadal men with confirmed low morning serum testosterone levels on at least two separate occasions. Exclusion criteria included women, pediatric patients, and men with contraindications to testosterone therapy such as prostate or breast cancer, untreated severe sleep apnea, or poorly controlled cardiovascular disease. The median age of participants was 56 years (range 29–74); 81% were White, 16% Black, 2% Mixed race, and 1% Asian, with 26% identifying as Hispanic [9]. Comorbidities were common, including obesity (70%), hypertension (50%), and type 2 diabetes (23%) [9].

The study duration was approximately 24 days, with the primary outcome being a proportion of men achieving a 24 h average serum testosterone concentration (Cavg 0–24 h) within the normal range (300–1080 ng/dL) at the final visit. Eighty percent of participants achieved this endpoint (95% CI, 72–88%). Secondary outcomes included the proportion of patients with maximum testosterone concentrations (Cmax) below predefined safety thresholds (1.5 times the upper limit of normal for total testosterone which is 1620 ng/dL), which were achieved in most patients [9].

Longer term follow up data is available from a 4-month uncontrolled ambulatory blood pressure monitoring study in 128 hypogonadal men (Study 18-001), which reported a mean systolic BP increase of 4.3 mmHg and a diastolic increase of 1.4 mmHg [9]. These findings are consistent with class-wide concerns regarding modest but rapid blood pressure elevations with oral TU.

The strengths of Tlando lie in its fixed-dose regimen, which simplifies therapy, improves adherence, and reduces the need for repeated titration or frequent laboratory monitoring. However, this can also be viewed as a limitation, as it does not allow for personalization of therapy, potentially leading to subtherapeutic or supratherapeutic levels in patients with variable absorption or comorbidities affecting pharmacokinetics. Furthermore, the trial’s short duration, open-label design, and absence of comparator arms limit the ability to contextualize efficacy and safety relative to placebo or other testosterone formulations.

Tlando provides a convenient, simplified oral testosterone therapy that restores eugonadal serum testosterone levels in the majority of men. Its fixed-dose design makes it attractive for adherence but less flexible than titratable formulations. Additionally, it shares the class risks like polycythemia and needs the same lab monitoring (hematocrit, lipids, PSA) and its clinical use requires ongoing monitoring for hypertension.

### 2.3. Kyzatrex

Kyzatrex is approved for TTh in adult men diagnosed with either primary or hypogonadotropic hypogonadism, following documentation of low morning serum testosterone on at least two separate days and the presence of clinical symptoms. The recommended starting dose is 200 mg orally twice daily with food, titrated between 100 mg once daily and 400 mg twice daily based on serum testosterone levels measured 3–5 h post-dose [8,29]. Kyzatrex has been approved in three dosage strengths: 100 mg, 150 mg, and 200 mg [8,29].

In a multicenter, open-labeled trial (Study MRS-TU-2019EXT NCT04467697) enrolling 155 hypogonadal men, Kyzatrex was evaluated over approximately six months (up to 180 days). The primary efficacy endpoint was assessed at day 90, at which 88% of participants achieved testosterone levels within eugonadal range (222–800 ng/dL), with continued follow up through six months for secondary safety outcomes [8]. Secondary endpoints included Cmax thresholds and safety outcomes.

Safety assessments showed modest hematologic and cardiovascular effects. Hemoglobin increases were seen in 4.5% of patients; hematocrit was not formally assessed [8]. PSA elevations occurred in 2.6% of patients, with 1.3% exceeding 4.0 ng/mL [8]. Hypertension was reported in 3.2% of participants, and mean systolic BP increased by 1.7 mmHg at 4 months and 1.8 mmHg at 6 months, with greater rises in men with baseline hypertension [8]. As with other oral testosterone formulations, ongoing monitoring of hematocrit, blood pressure, PSA, and lipid parameters is recommended.

From a practical and access perspective, Kyzatrex has unique considerations. Logistically, Kyzatrex is the simplest to obtain for patients compared to the other oral formulations as there is no insurance coverage and it is a self-pay only oral testosterone formulation. The cost is ~USD 150 which is not unreasonable. It is also beneficial for providers and practices as there is no prior authorization required. As with Tlando, the Kyzatrex trial is limited by its open-label, single-arm design, which limits generalizability and prevents direct comparisons. The Jatenzo trial, by contrast, included an active comparator arm, although it was likewise open-label and included no placebo arm.

Kyzatrex effectively restored testosterone concentrations to the eugonadal range in the majority of men with hypogonadism and demonstrated a generally tolerable short-term safety profile. It stands out most for being the most straightforward to obtain as prescribers and patients can bypass insurer denials with direct self-pay pricing.

### 2.4. Comparison of Kyzatrex, Tlando, and Jatenzo

**Figure 2:**
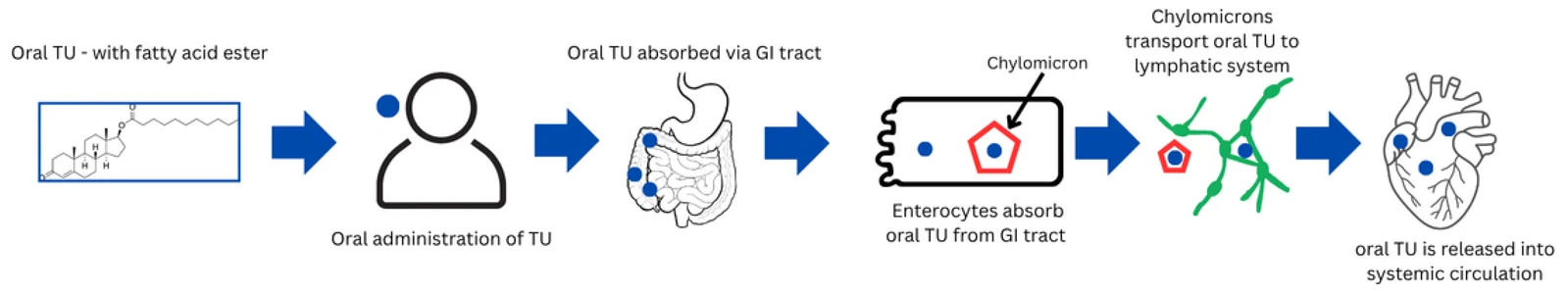
Oral TU absorption mechanism.

Kyzatrex, Tlando, and Jatenzo are oral testosterone undecanoate formulations absorbed via the intestinal lymphatic system, bypassing first-pass hepatic metabolism, as illustrated in Figure 2. Kyzatrex is taken with food to optimize absorption, with fat content markedly increasing exposure (area under the curve increased 37–97% vs. fasting); after 90 days, the mean concentration was 393 ng/dL and maximum concentration was 852 ng/dL [8]. Tlando’s bioavailability is also food-dependent but unaffected by fat content, though fasting reduces the maximum concentration and area under the curve by 65% and 38%, respectively; the mean average concentration was 476 ng/dL and maximum concentration was 979–989 ng/dL [9]. Jatenzo absorption is influenced by fat content (15 g fat meal decreases exposure by 25% vs. 30 g), achieving a mean average concentration of 403 ng/dL and maximum concentration of 1008 ng/dL after titration [31]. All share similar distributions (around 40% bound to sex hormone binding globulin), metabolism (ester hydrolysis, testosterone conversion to dihydrotestosterone and estradiol), and excretion pathways (primarily urinary conjugates) [8,9,31].

In the aforementioned clinical trials, eugonadal levels were achieved in 88% (Kyzatrex), 80% (Tlando), and 87% (Jatenzo) of patients, with Jatenzo also demonstrating improvements in psychosexual function and body composition. Safety profiles overlap, with hypertension, polycythemia, and lipid changes as the most common adverse effects. Blood pressure elevation is a class-wide Warning and Precaution; the boxed warnings for blood pressure previously carried by these products were removed by the FDA as part of a class-wide labeling change applied to all testosterone products. No clinically significant hepatotoxicity has been reported and gastrointestinal symptoms are generally mild.

Tlando’s fixed-dose regimen offers simplicity and may improve adherence, while Kyzatrex and Jatenzo’s titratable dosing allows individualized optimization. Market uptake and insurance coverage vary, with cost considerations likely to influence accessibility across products. Kyzatrex and Tlando are generally priced competitively, with average wholesale prices for a 30-day supply in the several hundred-dollar range, and both offer copay assistance programs to reduce patient costs [8,9]. Coverage for these are available through many commercial insurance plans and select Medicare Part D formularies, although prior authorization is common [9,29]. Jatenzo has historically been the highest priced formulation, with average wholesale prices often exceeding others [31]. Insurers frequently require prior authorization or step therapy, and manufacturer copay assistance is available for eligible patients [10,31]. A summary comparison is provided in Table 1.

## 3. Challenges and Future Directions

Although oral TU formulations represent a major advance in testosterone replacement therapy, several important challenges and unanswered questions remain. These must be addressed to clarify their long-term role in the management of symptomatic male hypogonadism.

### 3.1. Blood Pressure and Cardiovascular Safety

Across trials, oral TU formulations consistently demonstrate small but clinically relevant increases in blood pressure. Jatenzo raised mean systolic BP by 3–6 mmHg, with 7% of participants requiring antihypertensive therapy [31]. Tlando increased systolic and diastolic pressures by 4.3 mmHg and 1.4 mmHg, respectively, in Study 18-001 [9]. Kyzatrex produced more modest increases (1/7–1.8 mmHg), although it was greater in those with pre-existing hypertension [29].

More broadly, registry and post marketing data on oral TU show small to moderate BP elevations, which are likely to be clinically relevant in populations with baseline hypertension or cardiovascular disease [35,36]. For example, in a cohort of 737 men on TU (non-oral), progressive BP lowering over long-term follow up was observed, though this may reflect selection bias and confounding results; notably, these data offer a contrast to short-term trials of oral TU [37].

Testosterone therapy, more broadly, has complex cardiovascular effects. While some observational data and mechanistic studies suggest beneficial effects on endothelial function and atherosclerosis, the evidence remains mixed and context-dependent [38,39]. Several other mechanisms have also been proposed, including elevations in hematocrit, sodium and water retention, and stimulation of erythropoiesis. Erythrocytosis is a class-wide effect of TRT, mediated through stimulation of erythropoietin, suppression of hepcidin, and enhanced iron absorption [40]. Both Jatenzo and Tlando have been associated with hematocrit increases, and higher hematocrit has been strongly linked to blood pressure elevations [41]. Although this phenomenon has been most clearly observed with oral testosterone formulations, it is likely a class effect of TRT. The apparent absence of blood pressure changes in earlier studies of injectable or transdermal formulations may reflect limitations in monitoring, as prior trials rarely employed ambulatory blood pressure monitoring, a more sensitive method for detecting subtle increases [36].

These aforementioned physiologic mechanisms help explain the cardiovascular risk signals observed in clinical studies of oral testosterone therapies. Across randomized data, physiologic replacement has not increased major adverse cardiovascular events (MACE); however, non-MACE signals (e.g., atrial fibrillation, acute kidney injury, pulmonary embolism) were numerically higher with testosterone therapy [3,42]. In contrast, supraphysiologic exposure (e.g., non-medical anabolic–androgenic steroid use) is consistently linked to cardiomyopathy, arrhythmias, and thromboembolic events in observational cohorts and reviews supporting a dose-toxicity gradient [43,44]. Therefore, as cardiovascular risk appears to scale with exposure, supraphysiologic dosage is discouraged given absent clear benefits and notable risks [1,42,45,46]. By route, contemporary labeling and FDA communications indicate that testosterone products can raise blood pressure; among physiologic regiments, oral TU appears comparable to transdermal for MACE but merits monitoring of BP, hematocrit, and lipids given first-pass/hepatic and lipid transport considerations [42,47].

Preclinical and mechanistic evidence supports these clinical patterns. Animal models show testosterone increases erythropoiesis and sodium retention, enhances platelet thromboxane A2 release, and modifies lipid and plaque biology, improving endothelial nitric oxide signaling at physiologic levels but promoting LDL oxidation and inflammation at higher doses [40,48]. High-dose androgen exposure also induces myocardial and ion-channel remodeling consistent with arrhythmogenic risk [42,48]. While this offers biologic plausibility, translation to human outcomes is limited by species differences and supraphysiologic dosing.

Cardiovascular outcomes with testosterone replacement are most strongly influenced by dose/exposure, route of administration, and duration of therapy, with physiologic replacement regimens showing no increased risk of major adverse cardiovascular events (MACE), while supraphysiologic dosing and longer duration may increase some risks.

### 3.2. Comparative Effectiveness

No large, randomized, head-to-head trials compare oral TU formulations with injectable, transdermal, or implantable testosterone therapies. Thus, the relative efficacy and safety of oral TU remains uncertain. It is also unknown whether certain patient groups (e.g., men with poor adherence to injections, needle aversion, or variable absorption with gels) derive preferential benefit from oral TU. Direct comparative RCTs are needed, measuring not only biochemical endpoints but also sexual function, quality of life, body composition, and cardiovascular outcomes.

### 3.3. Absorption Variability and Non-Interchangeability

Despite bypassing first-pass hepatic metabolism, absorption of oral TU is variable, influenced by dietary fat, gastrointestinal physiology, and interindividual differences in lipid handling. Tlando’s fixed-dose regimen offers simplicity but may result in suboptimal or excessive exposure in some patients. Jatenzo and Kyzatrex allows titration, yet current algorithms may not fully account for this variability. Pharmacokinetic modeling studies could help identify predictors of absorption and support adaptive dosing strategies or next-generation formulations with more consistent bioavailability.

Additionally, non-interchangeability is a regulatory and clinical limitation. These products are not bioequivalent and cannot be substituted for one another. Each has unique pharmacokinetics, dosing, and titration protocols, and switching between them requires re-titration and monitoring of serum testosterone [8,9,31].

### 3.4. Symptom Outcomes and Quality-of-Life Measures

While oral TU trials consistently report biochemical normalization of testosterone, validated symptom outcomes are limited. Jatenzo demonstrated improvements in psychosexual function, but Tlando and Kyzatrex did not employ validated questionnaires [8,9,31]. Since symptom relief is the ultimate therapeutic goal, future research should incorporate standardized instruments such as the International Index of Erectile Function (IIEF) and Aging Male’s Symptoms (AMS) scale. This will allow for consistent, reproducible measurement of sexual function, global symptom burden, and energy/vitality [49].

## 4. Conclusions

Oral testosterone undecanoate formulations such as Kyzatrex, Tlando, and Jatenzo offer effective, non-invasive options for restoring eugonadal testosterone levels in men with symptomatic hypogonadism. These oral options fill an important niche for patients who cannot or prefer not to use injections or transdermal forms, expanding patient choice. Clinical trials demonstrate high efficacy and comparable safety profiles for all three agents. In practice, the choice between these therapies should be guided by individualized patient factors, including need for dose titration, adherence preferences, cardiovascular risk, and cost/insurance considerations. Tlando’s fixed-dose regimen may favor simplicity and adherence, while Kyzatrex and Jatenzo allow individualized titration to optimize serum testosterone levels. Finally, if insurance authorization denials is an ongoing battle, Kyzatrex does offer a reasonable self-pay only option for patients. Ongoing research will be essential to inform optimal monitoring strategies and refine their role in personalized management of male hypogonadism.

## Figures and Tables

**Table 1:** Comparison of Jatenzo, Tlando, and Kyzatrex.

Drug	Dosing Regimen	Absorption	Efficacy	Adverse Effects	Market Notes
Jatenzo	BID starting dose of 237 mg; titrated 158–396 mg BID based on serum T (6 h post-dose)	Oral; with food; 15 g fat meal decreases exposure by ~25% vs. 30 g fat meal	87%	Increase mean systolic BP + 3–6 mmHg, GI upset, polycythemia	First FDA approved oral testosterone undecanoate; established coverage patterns
Tlando	Fixed dose of 225 mg BID; no titrations	Oral; with food; unaffected by fat content but fasting decreases max concentration and area under curve	80%	Hypertension, polycythemia, lipid changes	Fixed-dose niche; variable uptake
Kyzatrex	BID starting dose of 200 mg; titrated 100 mg QD to 400 mg BID based on serum T (3–5 h post-dose)	Oral; with food; fat content increases area under curve 37 to 94% vs. fasting	88%	Increase mean systolic BP + 1.8 mmHg, polycythemia, edema, lipid changes	Newer entry; variable insurance uptake; reasonable self-pay option (USD 150)

## Data Availability

No new data were created or analyzed in this study.

## Abbreviations

The following abbreviations are used in this manuscript:

TTH	Testosterone therapy
