# Correction: Rosen, S.H.; Asanad, K. Treatment of Symptomatic Male Hypogonadism with New Oral Testosterone Therapies: A Comparative Review of Jatenzo, Tlando, and Kyzatrex. *Medicines* 2026, *13*, 1

**DOI:** 10.3390/medicines13040030

**Published:** 2026-10-23

**Authors:** Samantha H. Rosen, Kian Asanad

**Affiliations:** Keck School of Medicine of USC, University of Southern California Institute of Urology, Los Angeles, CA 90089, USA; shrosen@usc.edu

Error in Table

In the original publication [1], there was a mistake in Table 1 as published in the Adverse Effects column; the systolic blood pressure figure given for Jatenzo was inconsistent with the figure stated in the text of Section 2.1. In the Adverse Effects column, the systolic blood pressure range given for Jatenzo (3–5 mmHg) was inconsistent with the range stated in the text of Section 2.1 (3–6 mmHg); the value has been corrected to 3–6 mmHg to match the source publication. 

Text Correction

There were errors in the original publication regarding regulatory labeling, the design of the pivotal clinical trial, the attribution of efficacy data to their source studies, and the presentation of product pricing. Corrections have been made as set out below.

Regulatory Labeling (Section 2.1, Paragraph 3; 2.4, Paragraph 2)

The original text stated that Jatenzo carries a boxed warning regarding uncontrolled hypertension. The U.S. Food and Drug Administration removed the boxed warning relating to blood pressure from all testosterone products as part of a class-wide labeling change. The authors were not aware at the time of writing that this warning had been removed. A correction has been made to both sections as follows.

“The safety profile includes secondary polycythemia, peripheral edema, and dyslipidemia [31]. A distinctive concern is blood pressure elevation, causing mean systolic BP increase by 3–6 mm Hg, with greater rises in men with pre-existing hypertension, and 7.2% of participants requiring initiation or escalation of antihypertensive therapy during the study [33]. Under current labeling, blood pressure elevation is a Warning and Precaution rather than a boxed warning, Jatenzo remains not recommended for men with uncontrolled hypertension, and regular monitoring of hematocrit, blood pressure, PSA, and lipids is required [31].”

A correction has been made to Section 2.4, Paragraph 2:

“In the aforementioned clinical trials, eugonadal levels were achieved in 88% (Kyzatrex), 80% (Tlando), and 87% (Jatenzo) of patients, with Jatenzo also demonstrating improvements in psychosexual function and body composition. Safety profiles overlap, with hypertension, polycythemia, and lipid changes as the most common adverse effects. Blood pressure elevation is a class-wide Warning and Precaution; the boxed warnings for blood pressure previously carried by these products were removed by the FDA as part of a class-wide labeling change applied to all testosterone products. No clinically significant hepatotoxicity has been reported and gastrointestinal symptoms are generally mild.”

Characterization of Clinical Trial Design (Section 2.1 Paragraph 5, Section 2.3 Paragraph 4)

The original text described the Jatenzo evidence base as lacking placebo or comparator arms. The pivotal trial (CLAR-15012; NCT02722278), reported by Swerdloff et al., was a randomized, active-controlled, open-label study in which patients were assigned in a 3:1 ratio to oral testosterone undecanoate or a 2% topical testosterone solution as an active comparator. A correction has been made to Section 2.1, Paragraph 5:

“The pivotal trial (CLAR-15012; NCT02722278) was a randomized, active-controlled, open-label study in which patients were assigned in a 3:1 ratio to oral testosterone undecanoate (*n* = 166) or a 2% topical testosterone solution (*n* = 55) as active comparator [34]. Important limitations of the available data include the open-label design, the absence of a placebo arm, and the fact that efficacy was summarized separately by treatment group without formal statistical comparison between arms, which reduce internal validity and preclude precise attribution of efficacy or safety signals to Jatenzo alone. No large randomized, placebo-controlled trials directly compare Jatenzo to injectable or transdermal formulations, leaving its relative efficacy uncertain.”

A correction has been made to Section 2.3, Paragraph 4:

“From a practical and access perspective, Kyzatrex has unique considerations. Logistically, Kyzatrex is the simplest to obtain for patients compared to the other oral formulations as there is no insurance coverage and it is a self-pay only oral testosterone formulation. The cost is ~USD 150 which is not unreasonable. It is also beneficial for providers and practices as there is no prior authorization required. As with Tlando, the Kyzatrex trial is limited by its open-label, single-arm design, which limits generalizability and prevents direct comparisons. The Jatenzo trial, by contrast, included an active comparator arm, although it was likewise open-label and included no placebo arm.”

Attribution of Efficacy and Extension Data (Section 2.1, Jatenzo, Paragraph 2)

In the original text, the 4-month primary endpoint of the pivotal trial and the 24-month efficacy and extension enrollment data were presented sequentially in a manner implying a single continuous study. These figures derive from two distinct programs. A correction has been made to Section 2.1, Paragraph 2:

“The pivotal inTUne open-label trial (CLAR-15012) enrolled 166 hypogonadal men aged 18–75 years (mean age 56.2, 87% White) for an initial 4-month study period, with titration to maintain eugonadal serum testosterone concentrations. All patients were started on 237 mg twice daily, then titrated to 158 mg, 198 mg, 316 mg, or 396 mg twice daily [31,32]. At the 4-month primary endpoint of this trial, 87.3% of men in the oral testosterone undecanoate arm achieved serum testosterone levels within the eugonadal range [11]. Separately, in the CLAR-09007 program, a 12-month extension study was available to 129 eligible participants; 86 enrolled and 69 completed 24 months of uninterrupted therapy, with efficacy sustained over this 2-year period [33]. Beyond biochemical efficacy, Jatenzo demonstrated improvements in psychosexual function, measured by the Psychosexual Daily Questionnaire, a prespecified secondary endpoint, with gains in sexual activity, desire, and function sustained through 24 months in the extension program [33].”

The authors state that the scientific conclusions are unaffected. This correction was approved by the Academic Editor. The original publication has also been updated.
